# Anomalous Growth Rate of Ag Nanocrystals Revealed by *in situ* STEM

**DOI:** 10.1038/s41598-017-15140-y

**Published:** 2017-11-27

**Authors:** Mingyuan Ge, Ming Lu, Yong Chu, Huolin Xin

**Affiliations:** 1 National Synchrotron Light Source II (NSLS-II), Brookhaven National Laboratory, Upton, NY 11973 USA; 2 Center for Functional Nanomaterials (CFN), Brookhaven National Laboratory, Upton, NY 11973 USA

## Abstract

*In situ* microscopy of colloidal nanocrystal growth offers a unique opportunity to acquire direct and straightforward data for assessing classical growth models. Here, we observe the growth trajectories of individual Ag nanoparticles in solution using *in situ* scanning transmission electron microscopy. For the first time, we provide experimental evidence of growth rates of Ag nanoparticles in the presence of Pt in solution that are significantly faster than predicted by Lifshitz-Slyozov-Wagner theory. We attribute these observed anomalous growth rates to the synergistic effects of the catalytic properties of Pt and the electron beam itself. Transiently reduced Pt atoms serve as active sites for Ag ions to grow, thereby playing a key role in controlling the growth kinetics. Electron beam illumination greatly increases the local concentration of free radicals, thereby strongly influencing particle growth rate and the resulting particle morphology. Through a systematic investigation, we demonstrate the feasibility of utilizing these synergistic effects for controlling the growth rates and particle morphologies at the nanoscale. Our findings not only expand the current scope of crystal growth theory, but may also lead to a broader scientific application of nanocrystal synthesis.

## Introduction

In the field of nanoparticle research, considerable efforts have been made to synthesize colloidal nanocrystals with well-defined geometry. The ability to carefully tune these geometric properties could open the door to nanocrystals with unique optical properties and electronic structures suitable for a wide range of applications^1,2^. However, synthesizing nanoparticles with a specific size, shape, and topology requires the ability to manipulate growth kinetics as well as a more fundamental understanding of the growth mechanics of nanoparticles. Among the leading challenges is the ability to acquire reliable experimental data that can be used to visualize complex growth processes under *in situ* conditions. These types of experiments are typically performed by traditional ensemble averaging methods such as light absorption and scattering spectroscopy^3–6^. To date, developments in *in situ* electron microscopy have enabled the imaging of colloidal crystal growth in liquid^7^. In the *in situ* experiment, direct spatiotemporal tracking of single particles to quantify growth kinetics would present a significant advancement as it would enable research into fundamental nanoparticles growth mechanics.

Recently, *in situ* assessment of the synthesis of noble metal nanocrystals (e.g. Pt, Au, Ag) has prompted great interest not only because of their elemental simplicity as model systems for studying growth kinetics, but also because of their rich portfolio of controllable attributes, such as physical morphology, surface plasmonic frequency, and electrochemical catalytic properties^8^. Several experiments have demonstrated different approaches for controlling Pt, Au, and Ag nanostructures^8–10^. For example, Pt nanoparticles can grow into nanocubes, triangles, or more complicated shapes like octahedral or star-like shape by varying reagent concentrations, surfactant species, and reaction time^8^. Nanoparticles composed of other noble metals, such as Au and Ag, have shown a similar potential to have their morphologies and physical properties tuned.

Classical theoretical frameworks, such as the nucleation theory^11–13^ and the Lifshitz-Slyozov-Wagner (LSW) growth model, are widely adopted for guiding synthesis of nanomaterials^14^. Under those theoretical models, particles first undergo a nucleation burst phase before a sustained grow phase. The latter can be further classified into two categories, namely diffusion-controlled growth and reaction-controlled growth^14^. However, these models are oversimplified since they are based on fixed experimental conditions, which often lead to inaccurate predictions and warrant further corrections of the model^15,16^. Recent developments in *in situ* transmission electron microscopy (TEM) using liquid cells have made it possible to validate these classical theories with a higher degree of certainty^7,17–20^. For instance, Zheng *et al*., for the first time directly observed single colloidal Pt crystal growth in solution^18^. With TEM, they utilized an electron beam to reduce Pt ions and initiate particle growth. Remarkably, they illustrated a novel pathway of particle growth, achieving a monodisperse size distribution either by monomer addition or particle coalescence. *Woehl et. al*. further extended *in situ* TEM research to evaluate possible radiation effect of the electron beam^21^. They demonstrated that, depending on the electron beam intensity, growth of Ag particles can be either diffusion-controlled or reaction-controlled. Their work revealed that the growth rate within the diffusion limited regime follows a power law of $\bar{r}\sim {t}^{1/8}$ ($\bar{r}$ is defined as the increment of particle size), which was considerably slower than a growth rate of $\bar{r}\sim {t}^{1/3}$ as predicted by the LSW model. Though the exact reason for this deviation remains an open question, their research provides evidence of a complex interplay between the growth rate of crystals and their surrounding environment.

Here, we report that the growth rate of nanocrystals can be significantly higher than what is predicated by the LSW theory. We imaged *in situ* Ag particle growth in a solution using a liquid cell inserted in a scanning transmission electron microscope (STEM, FEI Talos F200X at Center for Functional Nanomaterials, Brookhaven National Laboratory). We found an anomalously high growth rate of $\bar{r}\sim {t}^{0.89}$ when Ag nanoparticles were grown on a Pt substrate in a solution containing a Pt precursor. Further, we present an interesting synergistic effect of the catalytic properties of Pt and electron beam radiation on the morphological evolution of Ag nanoparticles, promoting either enhanced growth or dissolution.

## Results and Discussion

**Figure 1:**
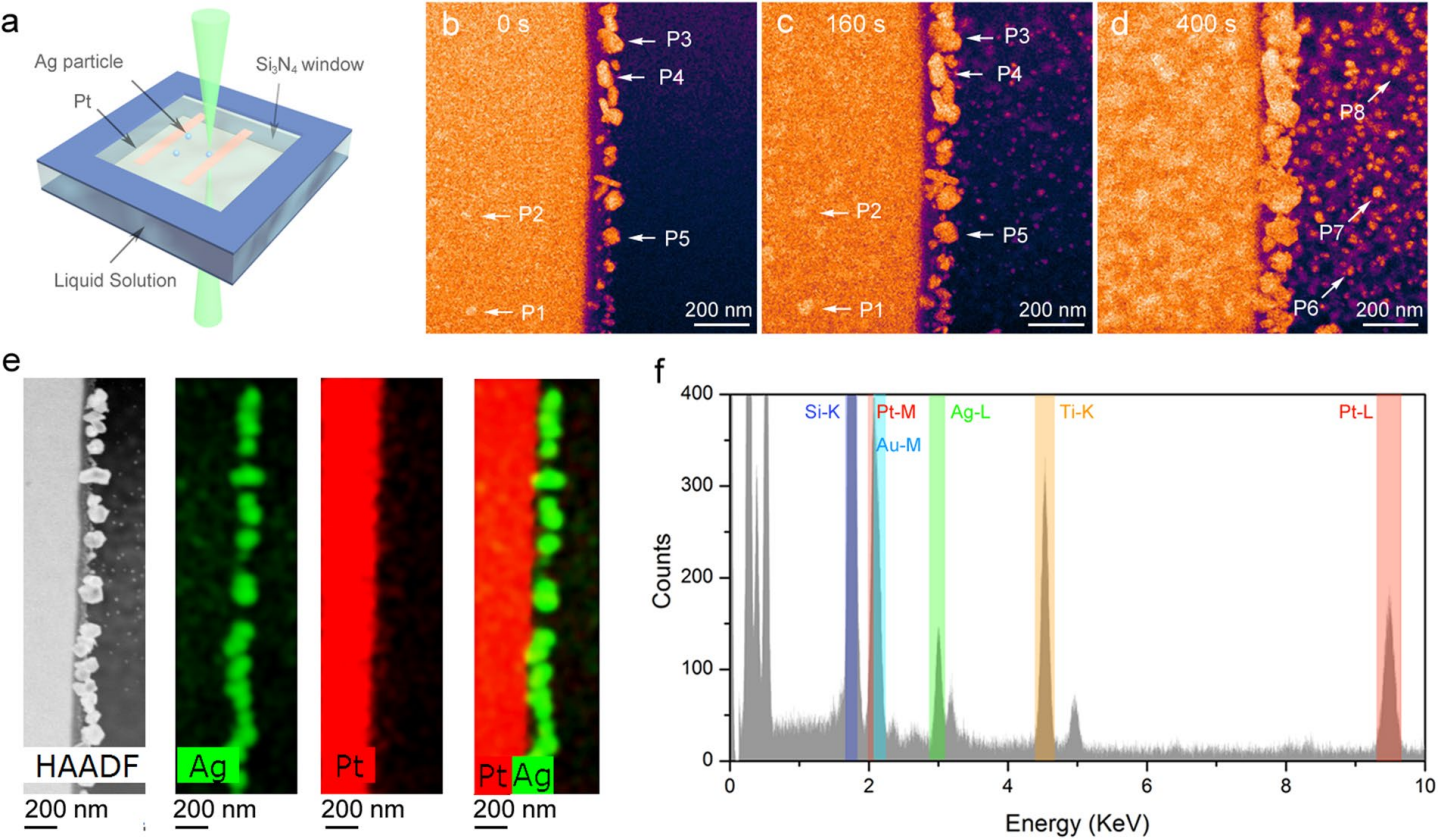
*In situ* investigation of Ag growth using a liquid STEM cell. (**a**) Schematic diagram of the liquid cell and experimental measurement geometry. (**b**–**d**) Real-time STEM images of nanoparticle growth in a solution (2 mM Na_2_PtCl_4_) at: (**b**) 0 s (**c**) 160 s, and (**d**) 400 s (**d**). Three groups of particles are indicated by the arrows: P1 and P2 are on the backside of the Pt bar; P3-P5 are on the edge of Pt bar; P6-P8 are in the nitride window region. (**e**) HAADF (high angle annular dark-field image) and elemental analysis derived from EDS spectrum (**f**) showing the Ag and Pt distribution maps of nanoparticles after 400 s.

Figure 1a shows a schematic diagram of the *in situ* liquid cell (Hummingbird Scientific). Two chips with silicon nitride windows (each 50 nm thick) were assembled to allow for direction observation of chemical reactions in the liquid cell. The thickness of the liquid layer was estimated to be around 1 μm. On the top chip, multiple Pt bars were pre-deposited on the silicon-nitride window, and a solution containing Ag nanoparticles and AgNO_3_ was then drop-casted on the inner surface of the top chip (See Materials and Method Section for details). Figure 1b is the false color STEM image showing the dispersed Ag nanoparticles before introducing the solution into the cell. All STEM images were taken at a beam current of 45 pA. Highly visible are the Ag nanoparticles attached to the edge of the Pt bar. A few of them are marked as P3 through P5. Though much less visible due to the strong attenuation of the electron beam, some Ag nanoparticles attached on the front surface of the Pt bar remained detectable (e.g. P1 and P2). The dark region on the right corresponds to an empty area of the nitride window, which is transparent to the electron beam. After initial measurements, we maintained a continuous flow through the cell (2 mM Na_2_PtCl_4_ in a solvent composed of water and isopropanol with a volume ratio of 1:10) and collected a series of STEM images at a 1 s interval.

At 160 seconds (Fig. 1c), we observed a substantial size increase of the initial Ag nanoparticles. These particles maintained more or less their initial shapes (e.g. P3 through P5). In the window region and on the Pt bar, we observed a growth of a large number of small nanoparticles. This observation was even more prominent after 400 s, as shown in Fig. 1d. A few of them are marked as P1, P2, P6, P7, and P8. All of the observed particles produced a uniform contrast in the STEM images, indicating that these are likely to be single-phase particles. This observation was confirmed by the energy dispersive X-ray spectra (EDS) measurement taken after 400 s, as shown in Fig. 1e and f. The elemental maps clearly show that those particles attached to the edge of the Pt bar are Ag nanoparticles. However, significant attenuation by the Pt bar prevents EDS measurements of Ag nanoparticles deposited on the back side of the Pt bar. It is also worth noting that there was no detectible Pt particle growth in the window region. One explanation is that the sizes of possible Pt particles were too small to be detected by STEM. For example, *in situ* observation shows that the Pt particles reduced by e-beam are typically saturated to a size less than 5 nm^18^. Another possible reason is that there exists some synergetic reaction between Pt (or Na_2_PtCl_4_) and Ag that prevents the growth of Pt particles. Further studies of the growth kinetics of the Ag particles can give insight into underlying physics of this phenomenon.

**Figure 2:**
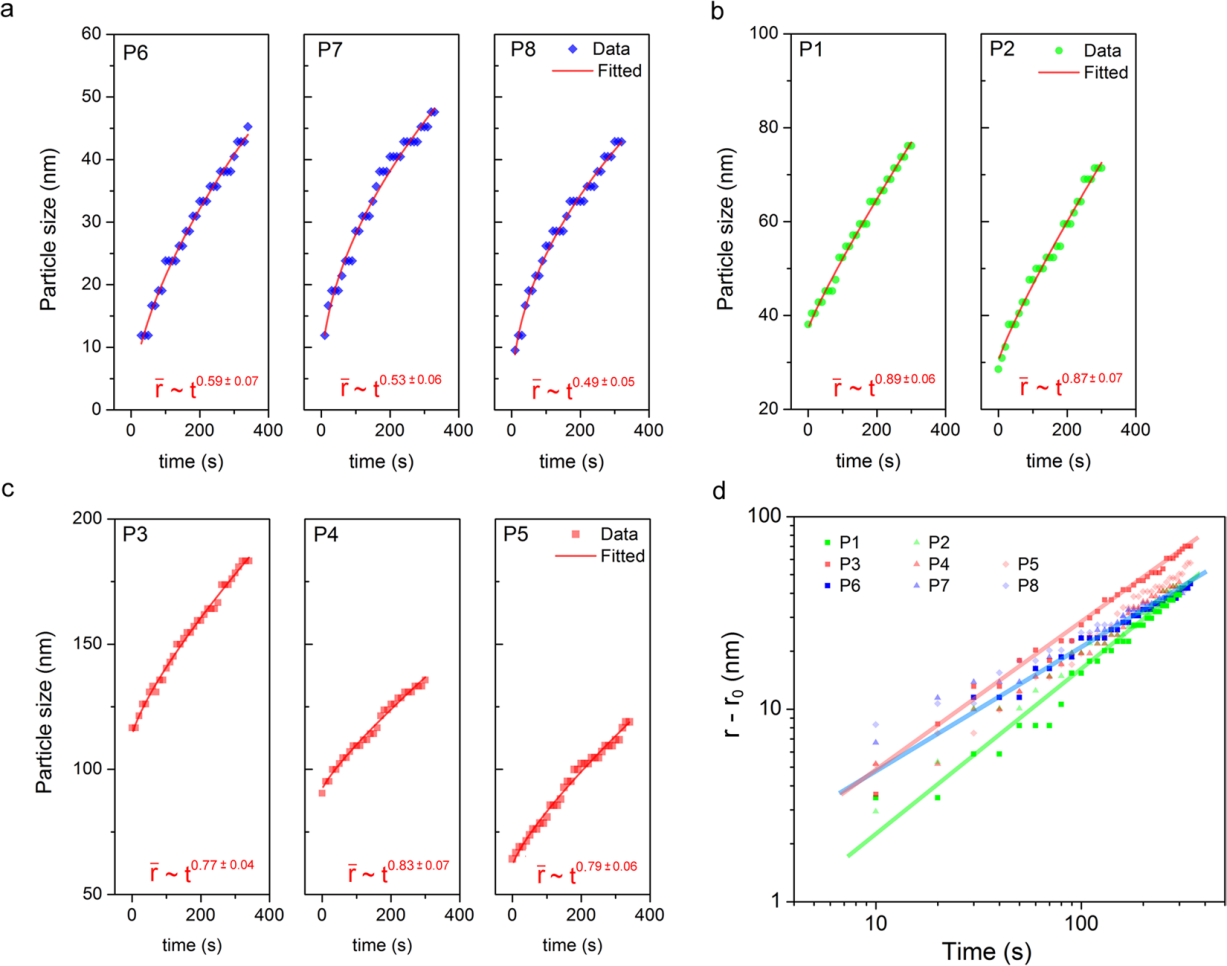
Size evolution of Ag particles. The growth curve is fitted as $r={{\rm{r}}}_{0}+{\rm{a}}\ast {{\rm{t}}}^{{\rm{b}}}$. The increment of particle size is defined as $\bar{r}\equiv r-{{\rm{r}}}_{0}$. (**a**) Particles on the window (P6, P7, P8, indicated in Fig. 1) showing a growth rate of  $\bar{r}\sim {t}^{0.5}$. The r_0_ is fitted to be 0.4 nm (P6), 5.2 nm (P7) and 1.2 nm (P8). (**b**) Particles on the Pt bar (P1, P2) showing a growth rate of $\bar{r}\sim {t}^{0.89}$. The r_0_ is fitted to be 37 nm (P1) and 30 nm (P2). (**c**) Particles at the Pt edge (P3, P4, P5) showing a growth rate of $\bar{r}\sim {t}^{0.8}$. The r_0_ is fitted to be 113 nm (P3), 91 nm (P4) and 61 nm (P5). (**d**) Log-log scale plot of particle size evolution showing the relationship of ${\rm{log}}({\rm{r}}-{{\rm{r}}}_{0})=\,{\rm{log}}({\rm{a}})+{\rm{b}}\ast {\rm{log}}({\rm{t}})$.

We recorded a time series of Ag growth in Movie 1 in the Supplementary material. Eight particles were randomly picked and analyzed for their size evolution over time (t). These particles were grouped into three categories: P1 and P2 are located on the back surface of the Pt bar (henceforth referred to as “on the Pt bar”); P3, P4, and P5 are at the edge of the Pt bar (henceforth referred to as “at the Pt edge”); P6, P7, and P8 are on the nitride window (henceforth referred to as “on the window”). The size evolution of these particles is summarized in Figure 2. As an evaluation of the kinetics of particle growth, particle size is fitted as $r\,={r}_{0}+a\ast {t}^{b}$, where r is the size of particle at time *t* (in unit of nm), r_0_ is the initial size of particle at time zero, *a* and *b* are the parameters need to be fitted, and *t* is time in unit of second. As shown in Fig. 2a, the particles on the window (P6, P7, and P8) exhibited a growth kinetic behavior where the particle radius r increases as $\sim {t}^{0.5}(\bar{r}\equiv (r-{r}_{0})\sim {t}^{0.5})$. According to LSW theory, a growth rate of $\bar{r}\sim {t}^{0.5}$ is a signature of reaction-controlled growth, while a growth rate of $\bar{r}\sim {t}^{1/3}$ indicates diffusion-controlled growth^14^. With sufficient amount of reducing regent in a bulk solution, growth rate of a noble metal particle is thought to be limited by how fast metal ions can diffuse to the particle surface. Interestingly, the nanoparticles growing on the window *appeared* to exhibit reaction-limited growth. Even more interestingly, the particles grown at the Pt edge (P3, P4, and P5) and on the Pt bar (P1 and P2) displayed even higher growth rates than the LSW prediction, approaching ${t}^{0.8}$ and ${t}^{0.89}$, respectively. Figure 2d plots the evolution of $r-{r}_{0}$ with respect to time (*t*) in a log-log scale. The slope of the curve indicates the magnitude of the growth rate, e.g. *b* in the formula $\,r\,={r}_{0}+a\ast {t}^{b}$. Visually inspection of the slope clearly indicates the growth rate of the eight particles ranks in the following order: (P1, P2) > (P3, P4, P5) > (P6, P7). The results presented here indicate that the metallic Pt is strongly influenced the growth kinetics of Ag nanoparticles. In order to understand the cause of the observed anomalous growth rate, a closer look at the electrochemical reactions in our system is required.

Electron beam illumination causes radiolysis of water, which creates an abundance of aqueous electrons (e_aq_^−^) and free radicals. In the presence of a metal precursor, aqueous electrons and hydrogen radicals reduce the metal ions to form metal atoms. The reactions can be described as the following electrochemical reactions^22^:

1 $${H}_{2}{O}^{\underrightarrow{{e}^{-}radiation}}\,{e}_{aq}^{-},H{O}^{\cdot },{H}^{\cdot },H{O}_{2}^{\cdot },{H}_{3}{O}^{+},O{H}^{-},{H}_{2}{O}_{2},{H}_{2}$$

2 $${M}^{+}+{e}_{aq}^{-},{H}^{\cdot }\to {M}^{0}$$

In our system, the following specific reactions, with respect to normal hydrogen electrode (NHE) potential^23^, can take place:

3 $$A{g}^{+}(aq)+{e}^{-}\leftrightarrow Ag(s){E}^{0}=0.7994V$$

4 $${[PtC{l}_{4}]}^{2-}+2{e}^{-}\leftrightarrow Pt(s)+4C{l}^{-}{E}^{0}=0.73V$$

5 $$A{g}^{+}(aq)+Pt(s)+4C{l}^{-}\leftrightarrow Ag(s)+{[PtC{l}_{4}]}^{2-}{\rm{\Delta }}{E}^{0}=0.0604V$$

Electrochemical reactions (3) and (4) describe reduction of Ag^+^ and [PtCl_4_]^2−^. With the abundance of electrons produced by the electron beam illumination, we would intuitively expect both reactions to take place, which would lead to formation of Ag and Pt nanoparticles. However, as indicated by the electrochemical reaction (5), Ag reduction will take place simultaneously with Pt oxidation, which leads to dissolution of the deposited Pt atoms back to the liquid phase. In other words, the deposited Pt atoms on the Ag particle surface provide abundant catalytically active sites with a reduced energy barrier onto which Ag atoms can nucleate. Concurrently, the transiently deposited Pt atoms are re-absorbed into the liquid phase. We have noted that the electropotential for reaction (3) is small, and therefore it leads to the small driving force for reaction (3). However, it is likely that the transiently deposited Pt atoms are highly reactive, which facilitates driving the reaction (3) to the right. We believe this reaction is responsible for the observed anomalous high growth rate of Ag. In addition, the electron beam illumination can cause even more dramatic enhancement of the growth rate of Ag particles in the region close to Pt bars and on the Pt bars. When illuminated by the electron beam, substantially more secondary electrons are scattered from the Pt bars than other regions of the sample. These secondary electrons will function as additional electron source to initiate water radiolysis reaction and generate addition aqueous electrons, which boost the growth of Ag particles and Pt clusters on Ag particles. Both pathways facilitate highly efficient Ag^+^ reduction and strongly enhance Ag growth rate. Another possible pathway that may affect the Ag particle growth rate is originated from the Cl^−^ ions in the system. It is known that Cl^-^ ions will selectively scavenge hydroxide radicals^24^, which could possibly inhibit oxidative etching of the silver and lead to the increased observed growth rate.

**Figure 3:**
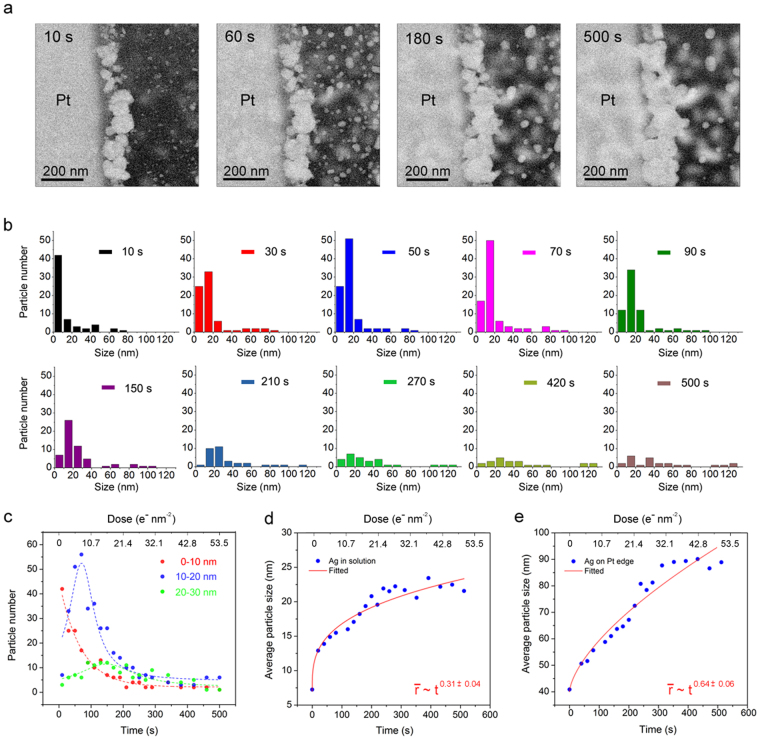
*In situ* inspection of Ag particle growth in isopropanol solution. (**a**) STEM images of Ag particle growth after 10 s, 60 s, 180 s, and 500 s. Beam current was 45 pA, corresponding to a dose rate of 0.107 (e^−^ nm^−2^ s^−1^). (**b**) Size distribution of Ag particles at different growth durations. All particles that are visible on the top chip are counted, and their size evolutions are measured manually. The error for particle size measurement is estimated to be 10% due to non-spherical shape of the particle. Initially (at 10 s), small size particles (0–30 nm) were observed mainly on the window; particles larger than 40 nm were observed mainly at the Pt edge. (**c**) statistical analysis of time evolution of particle numbers for particles with size of 0–10 nm (red), 10–20 nm (blue), and 20–30 nm (green). (**d**) Particle size distribution of initially small particles (on the window). (**e**) Particle size distribution of initially large particles (at the Pt edge).

To test this hypothesis, we conducted a second experiment by flowing the same solvent but without Na_2_PtCl_4_. For STEM imaging, all parameters were kept same, including the beam current 45 pA. Figure 3a shows the time series images of the particle growth. The data clearly showed that all the particles are growing in size, similar to the previous case. However, from a comparison of snapshots of the reaction at 60 and 180 s (Fig. 3a), we observed a reduction of the number of particles on the window. The evolution of particle size was more systematically seen from the particle size distribution shown in Fig. 3b. In the particle size distribution analysis, we only included particles that were near the top chip, which were in focus and showed sharp boundaries. The particles near the bottom chip, which were out of focus and had blurred boundaries, were not included. At 10 s, particle size distribution showed a large number of small particles (<10 nm), which were mainly located on the window. Large particles with size > 40 nm were found only at the Pt edge. With time, small particles gradually grew in size, as illustrated by the peak shift in the particle size distribution between 10 and 70 s. Starting at 90 s, we observed a clear drop in the number of particles with size below 30 nm. Close inspection revealed size-dependent growth behavior of the particles. Derived from particle size distribution data, we plot in Fig. 3c the temporal number distribution of particles with sizes below 10 nm (red curve), in a 10–20 nm range (blue curve), and a 20–30 nm (green curve) range, respectively. Within the first 100 s, a decrease in the particle number below 10 nm together with an increase in the particle number in 10–20 nm indicates a continued growth of initially nucleated small particles. In a simple system, where particles size increase is linear without the creation of new particles or annihilation of the existing particles, the temporal number distribution for particle size of 20–30 nm is a time convolution of temporal number distribution for particles with a size of 10–20 nm. Likewise, the temporal number distribution for 10–20 nm is a time convolution of the temporal number distribution for 0–10 nm. In our system, we found that the blue curve (10–20 nm) was roughly the shape of their curve (0–10 nm) convoluted with time (i.e. shifted in time). However, a steady drop of the green curve (20–30 nm) after about 150 s infers particle annihilation, an indication that 10–20 nm sized particles were either dissolved or incorporated into larger particles. The observed growth behavior is consistent with an Ostwald ripening process (LSW model) with a critical size around 20–30 nm.

Figure 3d shows the growth kinetics for the particles located only on the window. These particles exhibit an average size increase of $\bar{r}\sim {t}^{0.31}$, approaching to the theoretical growth rate of $\bar{r}\sim {t}^{1/3}$, corresponding to a diffusion-controlled growth as predicted by LSW theory. The observed difference in the two cases, with the Pt precursor ($\bar{r}\sim {t}^{0.5}$) and without the Pt precursor ($\bar{r}\sim {t}^{0.31}$), provides compelling evidence to support our hypothesis, where the transiently formed Pt atoms on the Ag nanoparticle surface provide an active site for Ag reduction, thereby enhancing the growth rate. It is important to point out that the observed growth rate of $\bar{r}\sim {t}^{0.5}\,$ does not follow the classical reaction-controlled growth. Instead, participation of Pt catalyzes the Ag reduction and substantially boosts nanoparticle growth. For this reason, we identify this growth modality as *catalyzed particle* growth. Movie 2 in the supplementary material records the particle evolution in real time.

Now, we turn our attention to the Ag nanoparticles grown on the Pt bar. As shown in Fig. 3e, the average particle size increased as $\bar{r}\sim {t}^{0.64}$. With the absence of the Pt precursor in the solution, the observed growth kinetics of $\bar{r}\sim {t}^{0.64}$ was lower than the previous case (see Fig. 2b and c). The observed decrease in the growth rate was expected since reactions (2) and (3) cannot occur without the Pt precursor in the solution. Nevertheless, the observed rate of $\bar{r}\sim {t}^{0.64}$ was still significantly higher than the rate for the particles grown on the window. This observation was also consistent with our proposed growth mechanism, where the scattered secondary electrons contributed to increased Ag growth rate.

**Figure 4:**
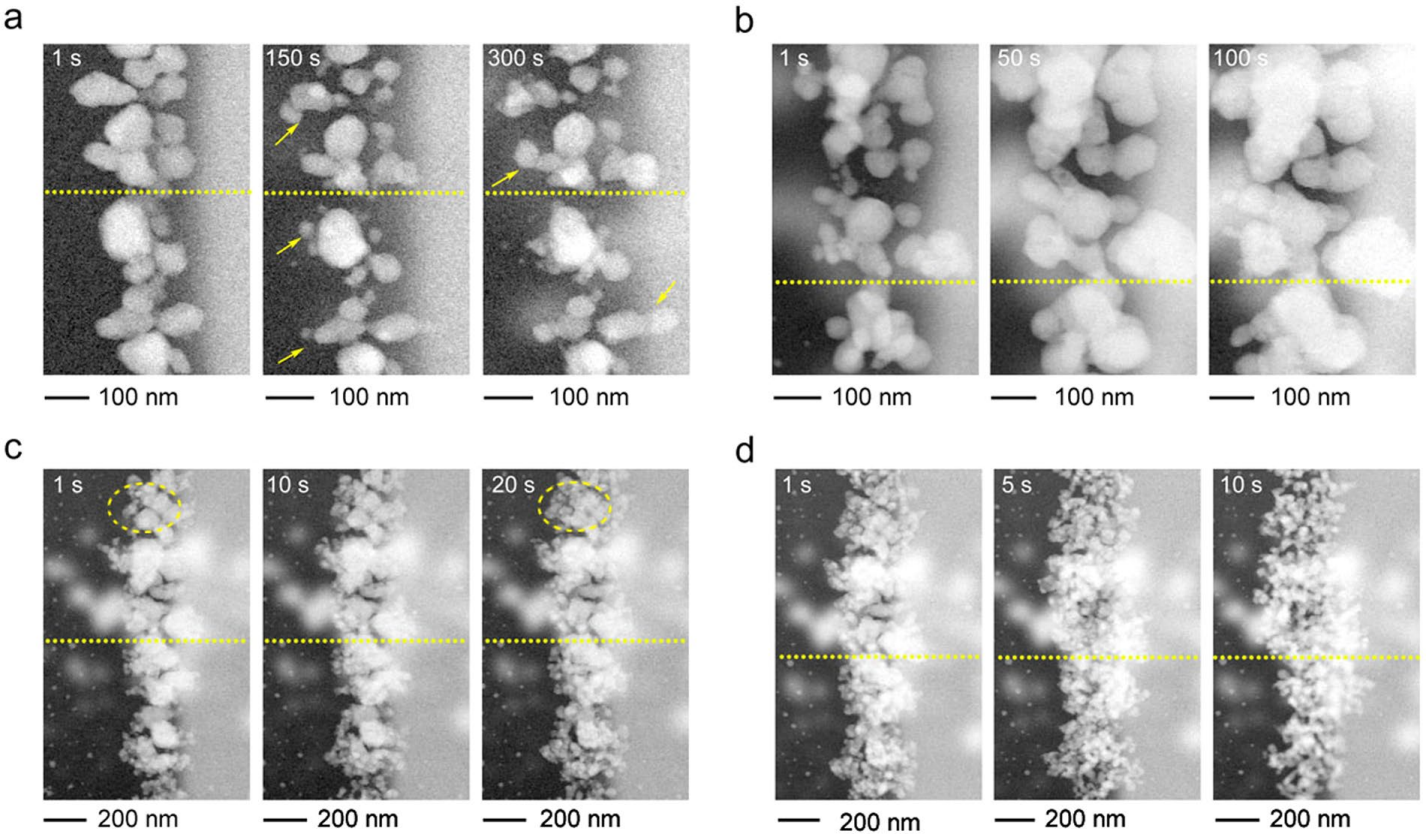
Effect of high Pt precursor concentration (20 mM Na_2_PtCl_4_) and high electron beam current on the morphology of the Ag nanoparticles. STEM images were taken over the same region of the sample with beam currents of (**a**) 45 pA (0.107 e^−^ nm^−2^ s^−1^), (**b**) 82 pA (0.195 e^−^ nm^−2^ s^−1^), (**c**) 236 pA (0.178 e^−^ nm^−2^ s^−1^) and (**d**) 426 pA (0.32 e^−^ nm^−2^ s^−1^), in sequence. The dashed lines provide a guideline to indicate the same location of particles in the images.

So far, we discussed the basic mechanism responsible for the observed anomalous growth rate of Ag nanoparticles. In our third experiment, we explored additional factors affecting nanoparticle morphology. Figure 4a shows the STEM images of Ag particle growth, with the same beam current of 45 pA and under a flow of 20 mM Na_2_PtCl_4_ solution, which is ten times the concentration used in the first experiment. After 150 s, we did not observe a noticeable particles size increase. Instead, we observed a significant change in particle morphology. As indicated by the arrows, the morphology of many particles evolved, branching out into smaller particles. This is direct evidence of mass redistribution. Redox processes, described by equations (1–3), are under dynamic equilibrium, where both growth and dissolution processes take place constantly at the interface between the Ag particle surface and the liquid solution. From water radiolysis, the reductant radicals such as H∙ and e_aq_^−^ expedite Ag growth, whereas the oxidant radicals such as HO∙ and HO_2_∙ facilitate Ag dissolution. The transiently formed Pt atoms provide more energetically favorable sites, on which the dissolved Ag^+^ ions can migrate and grow, leading to systematic mass redistribution to more energetically favorable growth sites. It should be noted that the local concentrations of the free radicals and the aqueous electrons are related to the electron beam intensity. Therefore, a higher electron beam current would result in a more accelerated mass redistribution rate. After another 60 s exposure at 45 pA (0.107 e^−^ nm^−2^ s^−1^), beam current was increased to and kept at 82 pA (0.195 e^−^ nm^−2^ s^−1^). Figure 4b illustrates the time sequences at 82 pA. The images taken at 1 and 50 s exhibited a faster growth of Ag nanoparticles with a more substantial mass migration. Figure 4c shows the image at a larger field of view. The beam current was increased to 236 pA, and the dose rate was estimated to be 0.178 e^−^ nm^−2^ s^−1^, which was close to dose rate used in Fig. 4b. As shown in Fig. 4c, we observed a significant amount of small Ag particles exhibiting dendritic growth, as indicated by the dashed circle. Further increasing the beam intensity resulted in a dramatic change in particle morphology. Figure 4d shows the image series taken at 426 pA (0.320 e^−^ nm^−2^ s^−1^). In Fig. 4d, the image taken at 1 s was taken instantly after switching the beam current to 426 pA, which showed a similar morphology to the image taken at 20 s in Fig. 4c. After 5 s exposure, a substantial decrease in the size of the Ag nanoparticles and fast growth of new Ag particles were observed. We hypothesize that a new dynamic equilibrium was established when we increased the electron beam current. Under these conditions, the Ag nanoparticles exhibit dendritic growth morphology, which is generally associated with fast growth rates^25^.

In conclusion, we have performed an *in situ* investigation of the growth mechanism of Ag nanocrystals in a liquid solution using STEM. We experimentally demonstrated nanoparticle growth rates significantly higher than predicted by classical growth theory. These growth rates can be explained by the catalytic properties of Pt and secondary electrons produced by the electron beam illumination. Further, we provide experimental evidence of how these effects can be used to achieve a wide range of controllable nanoparticle properties, such as growth rate, particle size distribution, and growth morphology. Two drastically different growth modes, conformational and dendritic growth, were achieved by varying the electron beam current. We believe that the presented mechanism for controlling growth parameters is based on the fundamental reactions at the electrochemical interface. Consequently, it is highly probable that the same approach can be used in other material systems. In addition, the diversity of particle morphology, as demonstrated in our work, may find applications for constructing templates for functional material systems. We believe that the presented results will significantly expand the current scope of crystal growth theory and find broad nanofabrication applications.

## Materials and Methods

### Synthesis of silver nanoparticles

Silver nanoparticles were synthesized according to methods found in the literature^26,27^. Specifically, 0.1 M AgNO_3_ aqueous solution was refluxed at 100 °C, followed by a quick injection of a second aqueous solution containing 2 wt% citric acid trisodium salt. After 5 min, the solution turned to greenish yellow color, and the reaction was stopped by cooling it down to room temperature. Then, the solution was stored in the dark until used for the experiments.

### *In situ* cell preparation

During the STEM experiment, the *in situ* environment was maintained using a commercial liquid cell (Hummingbird Scientific), which uses two silicon chips with a silicon nitride window (50 nm thick). The first chip, containing lithographically patterned Pt bars on the window, was treated with oxygen plasma to produce a hydrophilic surface. A small volume of a solution containing Ag nanoparticles and an additional amount of AgNO_3_ (~1 mM) was drop-casted on the chip and was allowed to dry at room temperature. The liquid cell was then assembled by stacking the first chip onto a second blank chip, creating a small space needed for holding solution. For the *in situ* experiment, the assembled cell was first plugged into the STEM without liquid. Solutions containing Pt precursor was then pumped into the cell at a flow rate of 2 µl/min. Since the gap between the two silicon nitride windows is very small (~1um), liquid cannot flow freely through the window region. Instead, the liquid sips into the window region by capillary force. When we flow the cell during the experiment, the liquid flows mostly through the liquid channel around the window region. Consequently, we expect that the total amount of silver remains approximately constant either in ionic or metallic state. The *in situ* STEM experiments were performed using a FEI Talos F200X STEM at the Center for Functional Nanomaterials of the Brookhaven National Laboratory. It is worth noting that our liquid cell holder has a notch on the chip clamp to increase the acceptance angle for detecting the emitted fluorescence X-rays from the sample. In conjunction with the four-quadrant detector geometry inside Talos F200X, a large fraction of fluorescence X-rays are collected directly by the detector without going through other parts of the holder, achieving excellent photon statistics.

### *In situ* STEM experiment

In the *in situ* experiment, solutions composed of isopropanol and water (10:1 in volume) with or without Na_2_PtCl_4_ were continuously flowing at a rate of 2 µl/min into the cell during observation. The imaging scanning rate is 1 s per image. Specifically, for the first experiment (Figs 1 and 2), 2 mM Na_2_PtCl_4_ was flowing into the cell. The beam current was 45 pA during imaging. The electron dose rate is estimated to be 0.107 (e^−^ nm^−2^ s^−1^). For the second experiment (Fig. 3), only pure solution (isopropanol:water = 10:1) was flowing into the cell. The beam current was kept at 45 pA. For the third experiment (Fig. 4), 20 mM Na_2_PtCl_4_ was flowing into the cell. Figure 4a was taken at a beam current of 45 pA, corresponding to a dose rate of 0.107 e^−^ nm^−2^ s^−1^. For Fig. 4b, the first image was taken after an additional 1 min exposure at 45 pA after the 300 s image in Fig. 4a. The beam current used in Fig. 4b was 82 pA, corresponding to a dose rate of 0.195 e^−^ nm^−2^ s^−1^. Figure 4c was taken immediately after Fig. 4b with a large field of view. The beam current was 236 pA, and dose rate was 0.178 e^−^ nm^−2^ s^−1^. Figure 4d was taken immediately after Fig. 4c with the same field of view. The beam current was 426 pA, and dose rate was 0.32 e^−^ nm^−2^ s^−1^.

### Particle size distribution

To evaluate the particle size evolution (Figs 2 and 3), we measured the particle size by manually drawing a line over the particles and measure the line length. Only particles that were present on the surface of the top chip were counted. As some particles are not spherical, we estimate the error in measuring particle sizes may be as much as 10%. It should be noted that we did not use software to automatically track particles and measure particle size, mainly due to the complex features present in the images. In Figs 2 and 3, particles form not only on the surface of the top chip, but also on the surface of back chip, which blurs the image and make automated particle tracking algorithms unreliable.

## Electronic supplementary material

Supplementary information

Supplementary Movie 1

Supplementary Movie 2
