# Supplementary material for: Anomalous Growth Rate of Ag Nanocrystals Revealed by in situ STEM
Source: Sci Rep. 2017 Nov 27;7:16420. doi: 10.1038/s41598-017-15140-y (PMC5703889; doi:10.1038/s41598-017-15140-y)
Supplement: Supplementary file 1 — Supplementary information [file 41598_2017_15140_MOESM1_ESM.pdf]

Anomalous Growth Rate of Ag Nanocrystals Revealed by in-situ STEM

Mingyuan Ge[1], Ming Lu[2], Yong Chu[1], and Huolin Xin[2*]*

[1] National Synchrotron Light Source II (NSLS-II), Brookhaven National Laboratory, Upton, NY 11973, USA

[2] Center for Functional Nanomaterials (CFN), Brookhaven National Laboratory, Upton, NY 11973, USA

STEM operation conditions: the liquid-cell imaging was performed in a FEI Talos F200X equipment with a X-FEG which is 4x brighter than a regular Schottky emitter. The condition of pixel dwell time = 2 us, HAADF inner semi-angle = 50 mrad, convergence semi-angle = 10 mrad, accelerating voltage = 200 kV was used for imaging. The dose rates and beam currents used for in-situ imaging are described in the figure captions in the main text.

SFig.1 shows the morphology of Ag particle growth after a prolonged reaction in the presence of Na_2PtCl_4. We can see that Pt is grown on the surface of Ag particle. We believed that after long time reaction with $AgNO_3$ being consumed out, $[PtCl_4]^{2-}$ ions start to be reduced to Pt particle without being oxidized back by Ag^+ ions.

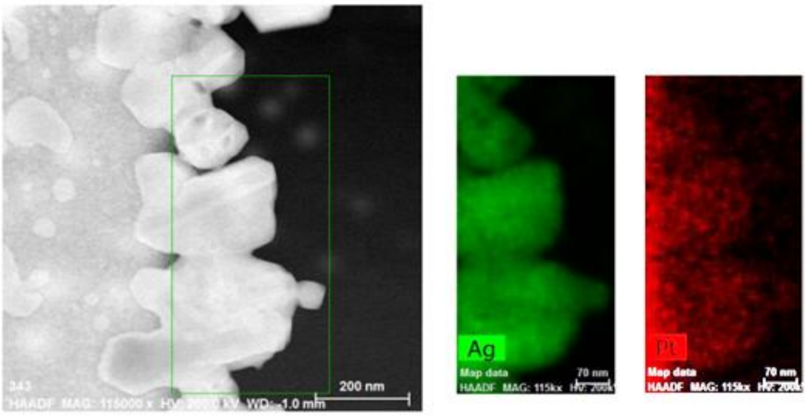

SFig. 1. HAADF image and EDS mapping of Ag and Pt after long time reaction.

Movie 1 records the real time evolution of Ag particle grown under the flowing of 2 mM Na_2PtCl_4 solution. Snapshots of the particle morphology were shown in Figure 1 in the main text. Movie is 50 x fast of real reaction.

Movie 2 records the real time evolution of Ag particle grown under the flowing of pure solvent (10:1 isopropanol and water). Snapshots of the particle morphology were shown in Figure 3 in the main text. Movie is 50 x fast of real reaction.
